# Understanding the Perspectives and Experiences of Employed Older People on Active Ageing in Their Later Working Life (Biographical Narrative Approach)

**DOI:** 10.3390/ijerph18136916

**Published:** 2021-06-28

**Authors:** Yong-Lim You, Hyun-Suk Lee

**Affiliations:** 1 Social Welfare and Counseling Department, Chodang University, Muan-gun 58530, Korea; ylyou@cdu.ac.kr; 2 Public Health Administration Department, Vision College of Jeonju, Jeonju-si 55069, Korea

**Keywords:** active ageing, older people, economic activity of older people, biographical narrative research

## Abstract

This thesis aims to understand the perspectives and experiences of older people regarding the concept of active ageing in their later working life with biographical narratives. This research adopted a biographical narrative interview for data collection. A total of 15 employed older people were interviewed by the researcher. The collected data were analysed using the biographical narrative analysis of Schütze (1983). The research findings are the following: in the first theme, the driving force to enable older people to choose active ageing in the workplace was their confidence in their work ability to include a challenging attitude at work. In the second theme, another driving force to enable older people to participate in economic activity that was considered was the individual workability of active older workers, including health rather than their chronological ageing in the labour market. However, in the third theme, research participants believed that the barrier of active ageing is a negative social prejudice on the working ability of older people. From this point of view, the research participants suggested that negative social prejudice for older workers should be overcome by active ageing experiences in age-friendly working environments as the fourth theme.

## 1. Introduction

People aged 65 and older now make up 14.9 percent of the Korean population, but the ratio will reach 20.3 percent in 2025 [1]. Accoding to an OECD report, the employment rate of older people over 65 is 31.3 percent in South Korea, the second among the OECD countries in 2019 [2,3].

In a survey by the Korea Labor Force Development Institute for the aged [4], 76.5% of the participants stated that their primary reason for working was to make enough money in order to raise their standard of living. In addition, according to a study on older people between the ages of 55 and 80 years, the main reasons for working were to provide a living wage (31.7%) and joy in working (20.4%). In a different study, the proportion of those who needed to work for economic reasons was 63% [5]. According to a national investigation into the daily lives of older people [6], it was shown that 69.9% of older employees went to work for economic reasons, 9.6% to maintain their health and 6.5% because they were happy to be at work.

Alongside the reliance older people on economic activity in a “Productive Welfare Regime”, which the President Kim Dae-Jung introduced to emphasize work in return for welfare [7], and the circumstances that the older population of Korea is rapidly increasing (since an increased older population negatively influences the income structure of older people), the government is developing employment policies and programmes for older people. 

As a step of the Productive Welfare Regime approach, in 2004, the Job Creation Act was developed in response to the view that the state should provide policies and practices to create jobs for older people in order to reduce the cost of social security. The Act was also designed to help older people achieve active later life through their employment. In particular, the Act aimed to prevent older people’s problems such as poverty, physical health problems and mental illness through active ageing [8].

From this perspective, the active ageing theory can reliably contribute to understanding the kind of ageing that my research group of employed older people is achieving and how it is correlatively influenced by economic activity. Active ageing is further reviewed in the literature review section of.

As it mentioned earlier, the Korean government has developed active ageing policies and programmes focusing on the Job Creation Act. Alongside the need of older people to work in the productive welfare regime, it can be assumed that health, social participation in particular, economic activity and living security in active ageing are interactively relevant for older Korean people. However, the more that older Korean people are healthy and secured in living, the more that older Korean people are interested in economic activity. In this context, it can be relevant for this research to understand the perspectives of employed older people on active ageing in Korea. Moreover, although the majority of older Korean people are involved in economic participation in active ageing, it is difficult to find research that listens to the voices of older people in depth in terms of economic activity in active ageing. 

In this sense, this research aimed to understand what brings economic activity in active ageing to older people, what tackles the economic activity of older people in active ageing and what the research participants suggest to tackle the barriers of achieving economic activity in active ageing in their biographical narrative contexts, which can provide a deeper interpretation of their narratives.

## 2. Literature Review of Active Ageing

The active ageing theory was introduced to tackle the disengagement theory approach, which theorized that older people are, in accordance with social isolation, indifferent and inhospitable [9].

The active ageing theory of Havighurst and Albrecht [10] also emphasizes the place of activity. This active ageing theory shows that the need for social participation and personal relationships deepens as people grow older. In addition, the more involved older people are in social participation, the more satisfied they are with life.

The proponents believed in active ageing, which emphasized social roles and social participation as the way to age well [11]. On the other hand, some researchers asserted that the active ageing theory was situated within the “busyness” of Western culture, as associated with capitalism, which abundantly expanded in the 1950s and 1960s. At the time, they conceptualized that older people tend to enjoy a retired life and live active and creative lives in a developed pension system [12]. 

In this context, international concepts of active ageing were defined by the OECD, EU and WHO. The OECD defines the concept of active ageing as “the capacity of people, as they grow older, to lead productive lives in the society and economy. Active ageing implies a high degree of flexibility in how individuals and families choose to spend their time over life—in work, in learning, in care-giving” [13]. The concept of active ageing, as defined by the EU, is “preparing for longer, more active and better lives, working longer, retiring more gradually and seizing opportunities for active contributions after retirement are the best ways to secure the maximum degree of self-reliance and self- determination throughout old age. This is true even in the face of fading faculties and growing dependency” [14]. The World Health Organization [15] also defined active ageing; “Active ageing applies both individuals and population groups. Active ageing allows people to realize their potential for physical, social, and mental well-being throughout the life course and to participate in society according to their needs, desires and capacities, while providing them with adequate protection, security and care when they require assistance”.

As shown above, each organization has their own approach to the definition of active ageing. When the EU and OECD emphasize the economic and social participation of the older people, the WHO underlines the social aspects of health and quality of life.

Walker [16,17] also pointed out that the EU has principally developed active ageing, which stresses participation and well-being in later life, for decades. 

Other researchers also claim that interrelationships in social roles and efforts to maintain one’s role in society can contribute to active ageing. According to their research, older people were likely to have better mental and physical heath when they had “the higher socioeconomic status of being employed”. However, they were unlikely to be wealthy enough to have an important social status, even though the intensity of this relationship tends to depend on the individual’s “life course and life situation” [18,19,20].

Nevertheless, Walker [9] argues that active ageing is a social matter, not an individual responsibility, because the barriers to active participation in employment are structural obstacles.

Taylor [21] presented another point of view for active ageing beyond the importance of employment in active ageing. He asserted that it would be significant to legislate longer working policies to reduce the social welfare costs raised by the ageing society. However, he argues that we should not overlook that retirement successfully helps older people to improve their health and quality of life. In addition, it should not only be the employment of older people that is excessively emphasized, but the risks that seniors may encounter in workplaces should be considered also. Furthermore, other social activities beyond employment must not be undervalued.

In this context, Clarke and Warren [22], extended the concept of the active ageing. They believed that active ageing should also regard everyday activities rather than just formal ones because older people are interested in their ordinary needs for health, deeds for daily life, events of the years and relationships with family and friends in their research findings. 

In the expansion of the active ageing definition, Bowling [23] explored the perception of older people in Britain. In the research findings, 43% of the people think that active ageing is “having or maintaining physical health and functioning”; 34% of older people consider active ageing as “leisure and social activities”; 18% of the research participants considered active ageing to be “mental functioning and activity”, while 15% of the respondents supposed that active ageing is “social relationships and contacts”.

On the one hand, alongside the Western approach to active ageing, Asian studies on active ageing have been performed. Zaidi and Um [24] devised a new Asian Active Ageing (AAII) that revised the active ageing index developed by the EU. Comparing the active ageing index of Asian countries, Thailand is higher than Indonesia. The two countries are more indexed than many European countries. Japan has the highest activity index, being comparable to Scandinavian countries. Older people were both physically and mentally active when they had a low pension income level, which can be the cause of a high employment rate and can fathom their family’s high informal income support levels in Thailand and Indonesia. 

Additionally, Wongsala, Anbäcken and Rosendahl [25] (2021) asked seniors in Thailand about the active ageing that the WHO defined with health, social participation and living security. They found that health plays a safe role in the disorders of everyday life caused by ageing. In addition, social participation, including volunteering, provides seniors with respect, maintains a social support network and makes their life meaningful and respected. Living security was recognized as “manageable living conditions” and “life well” in the balance between dependence and independence from children, while maintaining the “traditional value of gratitude” among generations.

According to Korea’s Active Ageing Index (AAI) research, the level of active ageing in older Koreans was higher than the average AAI (33.9) in all EU countries (33.9), but lower than China (37.3). South Korea was the 11th country, behind Germany. In addition, among the active ageing index in South Korea, the “social participation” and “independent, healthy and secure living” levels were below the employment level. The researcher assumed that the higher level of employment in AAI might be caused by the low pension income level.

From the Asian studies above, it can be assumed that Asian countries, including South Korea, tend to have a higher employment level than other countries because of a low pension income level. This research result seems to be unfortunate for the Asian ageing society. 

In Korea, many of the existing studies also discussed the WHO’s active ageing in active ageing research. Yoo [26] found that health, social participation and living security, which the WHO composed for active ageing, should be balanced to promote their life satisfaction.

Furthermore, Hyun [27] (2020) presented how active ageing factors have an impact on the life satisfaction of older people. Older people perceived health status, in their own view, as the most significant factor for their life satisfaction, while their participation and living security levels in active ageing influenced their life satisfaction relatively less.

Remarkably, many other researchers revealed that one significant factor of active ageing index was relevant for their later life. First of all, in Oh’s [28] research to understand the active ageing of older women who experienced bereavement, health was the most important factor that the research participants achieve active ageing in their daily life. In addition, younger adults tended to work more actively when they are healthy. Gi [29] emphasized the voluntary activity of older people in active ageing, while pointing out that Korean policies and practices for older people are placed in economic activity to ensure income security. Heo [30] revealed that the more public benefit, earned income and asset income increased, the more the activities of older people for active ageing increased. Additionally, Byun [31] showed that seniors aged 65–74, in the circumstances where economic and mental stability was secure, were more involved in employment and social participation.

In particular, for the younger-old, aged 50 to 64 years, Joo and Shin [32] (2020) analysed active ageing policies and programmes. This included the 50 Plus programme. They concluded that the policies and programmes centred on job creation, social participation and later life plan, but the latter was withdrawing the identity of older people.

## 3. Aims and Design

This thesis aims to interpret aspects of active ageing and the later working life of older people in South Korea in their own life stories and experiences. Fraser [33] supports the use of a biographical method for research purposes, remarking: 

“The identities and needs that the social welfare system creates for its recipients have always interpreted their identities and needs”.

In other words, the perceptions, thoughts and needs of older workers who are the recipients of a social welfare system, such as social policies and programmes in terms of older people, are interpreted versions of their identities and needs. Thus, a biographical method is adopted here to understand the interpreted identities and needs of older workers. This method offers individuals the opportunity to narrate life experiences and stories that serve as resources for a deeper interpretation. In particular, Hazan [34], Midwinter [35], Bernard and Meade [36] and Clarke and Warren [22] believe that it is significant, in research on older people, to listen to their life stories and experiences as influences on their later life circumstances. Therefore, the researcher who interprets an understanding of older people to include ageing and their later working life is recommended to listen to the life stories and experiences of older narrators. 

My respondents were all people over the age of 65, and thus defined by chronological age as older people because it is on the basis of chronological age that society defines older people.

## 4. Method

This research used a biographical-narrative interview to produce the life story of its narrator. A series of unstructured interviews was conducted. The interviewer did not interrupt the interview, but, rather, listened to the interviewee’s story with empathy. In particular, the biographers were able to construct their biographical narrations in their own way and according to their own preferences. In this way, 15 older workers who are over the age of 65 took interviews over three months. In this context, the following Table 1 summarises the personal data of the research group.

### 4.1. Data Collection

Purposive and theoretical sampling approaches, rather than random selection, were adopted. The sampling plan was grounded in the purpose of the research and the intention to reveal and understand older workers’ perspectives and experiences on active ageing. This research includes subjects recruited from a range of agencies of different sizes, of different industrial sectors and geographical localities. It uses material from interviews with older workers who belong to the ageing workforce (see the above table, Table 1).

The interviews were performed with 15 employees who were active in the Senior Clubs and Senior Citizens’ Association. The Korean government gave a contract to provide employment services for older people to Senior Clubs and the Senior Citizens’ Association, voluntary agencies that have been developed for older workers, while the government themselves concentrated on policies that would ensure employment for them. This was a major factor in the decision to select them as the organisations from which to recruit interviewees, to ensure that people with a wide range of views and positions were included and interpreting the current policies and practices of the South Korean government as an approach of active ageing.

The geographical sampling areas were Seoul, Inchon, Kwangju, Junju, Seongnam, Namwon, Gaphyung, Yeoju and Wanju. Seoul was selected for three reasons. Firstly, the administration of the whole country is centralised on Seoul. Secondly, it is the biggest city in Korea. Finally, the cities were chosen because they are likely to have the widest recruitment opportunities for older people, as a result of the first and second reasons. Inchon, Kwangju, Seongnam and Junju, cities ranging from medium to large populations, are included within the research to include a range of industries. Further to this, Namwon, Gaphyung, Yeoju and Wanju were chosen to explore the characteristics of small city workplaces for older people. In this context, it was expected that the Senior Club and the job centre of the Senior Citizens’ Association in each of them would provide a different set of characteristics from the rest and might help to address the research questions relating to older people’s employment in active ageing alongside more diverse types of jobs.

An interviewer listened to their stories and responded to what they were saying with respect, without disrupting their narration once they had started. Questions were asked only when they had finished telling their life stories. The interviewees were encouraged to feel that they could talk about their lives openly, even though most of them were shy to tell their life stories at first. However, at the end of interviews, people were happy to talk:

“I have got too many stories. I could write books about them” (from the research interviews).

Firstly, the biographical interviews were conducted and, secondly, the main research questions asked were as follows:

1. What brings you economic activity in active ageing?
2. What tackles your economic activity in active ageing?
3. What do you suggest for tackling the barriers of economic activity in active ageing?

However, in selecting employed older people, there is a limitation in this research. This research could not consider gender and education level for research sampling. This research has 10 female and 5 male research participants. It implies that the research could not show the balanced perspective of men and women. However, this research method adopted the biographical narrative approach to understand and interpret the research participants’ narratives in their individual life contexts. It means that this research has not concerned the general view of gender, but rather has considered the individual perspective on active ageing in working life. In this context, education level could not also be considered in this research sampling. There is only one research participant with a PhD, while there are four interviewees who have no educational experiences and four research participants who have primary school experiences. Clearly the employed older person with a PhD is a significant worker and could reveal an entirely different view of older workers in the research. In this sense, it can be difficult to show the identical perspectives of interviewees on active ageing in education level. However, as it mentioned earlier, this research method is a biographical narrative approach to deliver the voices of research participants in their individual life contexts. Thus, this research mainly attempted to understand each research participant’s narratives and experiences and interpret the meaning of their narratives in their individual biographical contexts.

### 4.2. Data Analysis

The collected data were analysed using the method provided by Schütze (1983) [37]. Schütze’s biographical narrative analysis method was designed to view narrators’ life stories to “reconstruct” the narrator’s biography. Each tape was listened to twice: the first time attempted to catch the sense as a whole and record impressions of the interviewee’s narrative and also of the interviewer. This helped to prepare the approach of the second interview. The second time of listening, with repetitions at every stage, was used to capture all the details on the transcription form that were to be used during the analysis. The transcript was written in Korean at the first stage. However, at the final stage of analysis, the quoted words of interviewees were translated into English to support the themes. At this stage, the three experts including the researcher were involved in translation work. One expert is English, who was completing a PhD course. The other is a Korean PhD student who has lived in the UK for over 20 years. Three experts discussed the translation of the Korean words of the interviewees into English words in order for the quoted words in this English thesis to imply the closest meaning of the narratives and experiences of Korean research participants.

The analytical method of “Biographical narrative analysis” (Schütze, 1983) [37] was selected and used for the analysis of the interviews. In particular, the biographical data were analysed according to Schütze’s three steps. Schütze uses the “biographical method” approach because he believes that biographical narrative interviewing is the most relevant approach to interpretive social research, as a result of the research, from the actors’ subjective perspectives and experiences, which form social reality for them (Schütze 1983) [37]. He develops biographical narrative analysis methods into a systematic procedure, employing three steps. These are made up of “formal textual analysis such as thematic segments, structural description of these segments and analytical abstraction” and the aim of the steps is to generate a theoretical model. Significantly, in the theoretical analysis stage, the researcher offers some implications and interpretations in terms of the perspectives and experiences of employed older people on active ageing in their later working life within biographical analysis (Schütze 1983) [37].

### 4.3. Ethical Issues

Klockars [38] and Reiman [39] concluded that it is very important that any kind of interview research should consider the ethical implications. Creswell [40] and Seale [41] stress that the key ethical issue is the consent of the interviewees. Their standards highlight the importance of allowing opportunities for interviewees to refuse or agree to participate in the research, which is achieved by frequently asking for their consent throughout the interview. Naturally, the same was applied to this research—before any interviews took place, the interview consent form was presented in Korean in order to obtain the consent of interviewees. A very gentle approach was used to obtain their permission to study them before they completed the interview consent form. Throughout the interview, the research issues in terms of the thesis were reiterated and the interviewees were questioned regarding whether they felt comfortable talking. As Glesne and Peshkin [42] suggest in their principles for ethical research, the interviewees’ names were made anonymous to ensure confidentiality, given that they did not want their identity to be known. With regard to the permission to record the interview, some of the interviewees felt uncomfortable, particularly with their voices being recorded. This can be reasonably understood in the Korean culture, as people have experienced military dictatorships that restricted the freedom of speech. The interviewer explained how important it was to record the interviewee’s voice and the confidentiality was promised by agreeing to destroy the data after writing up the thesis. After an interview, the interview transcript was shown to the interviewee, and they were given a final opportunity to agree to use their narrative. The transcripts that interviewees did not give consent to were not used.

### 4.4. Trustworthiness

Validity and reliability were established by positivists, according to Guba and Lincoln [43] (1985). These authors (1985) point out, however, that interpretivists should still consider validity and reliability, despite the fact that the measures used to answer these principles differ in qualitative research. They suggest the following four criteria for determining “trustworthiness” in the pursuit of naturalistic studies for this purpose:

- credibility (in preference to internal validity);
- transferability (in preference to external validity/generalizability);
- dependability (in preference to reliability);
- conformability (in preference to objectivity).

In this sense, throughout the data collection chapters of this study, the methodological definition has been constructed with honesty and scope.

## 5. Findings

The first theme is “5.1. The confidence of the employed older people on work ability and their challenging attitude at work led them to active ageing in employment.”. The second theme is “5.2. Considering individual work ability of active older workers in-cluding health”. The first and second theme are the driving force to lead older people to participate in economic activity. The third theme is “5.3. Negative social prejudices for the work ability of older people have been obstacles towards active ageing”. It pre-sents the obstacle of active ageing. The fourth theme is “5.4. Society’s negative preju-dice for older workers could be overcome by experience with active older workers in age-friendly working environments.”. It is the suggestion of older workers for tackling the barriers of economic activity in active ageing (see Table 2).

### 5.1. The Confidence of the Employed Older People on Work Ability and Their Challenging Attitude at Work Led Them to Active Ageing in Employment

The interviewees believed that professional workability allowed them to participate in their working places even though they feel weakened in strength. Furthermore, they were confident with their highly skilled work in an area that suited their workability. In this regard, they do not accept their chronological ageing, but rather they challenge the ageing and attempt to realize their potential including in employment. As a result, they are able to receive praise from their supervisors and respect from their co-workers.

#### 5.1.1. Deteriorating Health but Professional Work

In the interviews, it was apparent that even these older people have a negative perception of ageing. Most interviewees thought that ageing shows in the loss of physical strength and in poor eyesight, but this is thought to have little influence on their capacity. Interviewee C believed that ageing makes her feel tired and at the beginning of her narrative she concentrated on the physical effects of ageing. Yet, when she talked about her performance, she confidently said,

“God has given me enough health to do this job”.

In this narrative, the interviewee is working. The reason why she is assured about her work performance, in contrast to the physical consequence of her feeling tired, is God, who sends her the health to work well. Although she feels that she has good health and high workability in comparison to other older people, she says she is aware of her deteriorating health. When she was younger, she did not often feel too tired. If she slept well all night, by morning, she always felt refreshed. However, nowadays, she felt tired in the morning despite a good night’s sleep. She tended to droop and complained about her nagging feeling of being tired. In this sense, the increase in physical tiredness was natural for her, but interestingly, she could not tolerate mistakes in her work; it must always be carried out well. Her present employer complimented her on her performance, as her previous employer used to. She also emphasized that she works as hard at present as she ever did.

“I have no time even to go to the toilet because I am too busy completing the orders”.

In particular, “completing the orders” indicates her general sense of duty. Furthermore, her outlook has practically influenced a way of life in which she does her best to complete orders even when it leaves her no time to go to the toilet, which is often the most urgent physiological need. More significantly, even the conflict between her outlook and a situation that precluded meeting her most urgent personal needs did not make her give up her work. Instead, in her terms, spiritual power led her to resolve the conflict and realize her values. In other words, despite deteriorating health, spiritual strength made her reach the belief that she can work well.

#### 5.1.2. Highly Skilled Work in Her Job Area

Interviewee E also said that ageing could affect the physical strength of older workers. For instance, she felt less able to carry heavy things. However, she pointed out that she could.

“…work better than younger workers here. Only I feel a little less physical strength compared to when I was younger. I am still highly skilful”.

Her concern is her good workability rather than ageing. She believes that her workability is better than the workability of younger people. She is, however referring to her current workplace—she compares this with past situations. She used to work on construction sites. As an older worker she worked in a food delivery service centre, a take-away restaurant. She has had two different types of job, in different settings. However, neither of these places can be easy for everyone to work in. When she compared her work in the past and now, she thought that her two jobs have called for different degrees of strength. Interestingly, however, she believes that her work performance in her current job is better than in her previous job, although she agrees that her physical strength is declining. The situation that she is facing is paradoxical. She has a positive view of the better workability of older employees but is also trying to accept the loss of the physical strength she once had. She tries to explain the paradox by referring to her excellent skills such as cooking skills or managing delivery food services, which may prove to her that her work performance has not deteriorated, and she can still trust in her workability, despite a little downturn in physical strength. This may be her subjective assessment, but it also seems that her strong confidence underlies and supports her good work performance. Furthermore, her faith in her good work performance reflects her motivation to achieve active ageing in work.

#### 5.1.3. His Sense of Challenge to Work

Interviewee M has an individual positive and active approach to the concept of ageing. He thinks that ageing does not affect the sense of challenge to let him to perform actively at work.

“So far, it is no problem at all to work. In particular, in the sense of challenge, growing older doesn’t change us!”

“Ageing affects our working. Eyesight gets worse and hearing gets weaker. I don’t hear well, particularly when I’m on the phone. However, different people have different levels of strength. Anyway, now, I don’t feel as strong as when I was young. But I can keep up with younger workers in work performance. However, I feel tired a lot. It is hard work carrying heavy loads in my deliveries”.

In his comments about his perception of ageing, he used the words “keep up with”. These words show his attitude to both ageing and working. He denied the gap between younger and older people in work performance, which is the most important attribute in the employment market and also claiming that there is no difference between the work performance of younger people and older people. His perception of ageing appears very candid and also challenging. It is suggested that his perception derived from his experience of life, which has not been easy. He began after the Korean War, walking as much as 12 km every day to school, when the bombed routes prevented the trains and buses from running. In addition, his family was so poor that as soon as he finished secondary school he had to begin work. He has never stopped working since those days. However, he boasted about his children’s success. He was proud that his two children had graduated from prestigious universities and obtained good jobs, thanks to his hard work. In this context, it is understandable that he welcomed the challenge of work and was confident that he could keep up with the work performance of younger workers by overcoming his physical weaknesses and problems with eyesight and hearing. As he has lived a challenging and harsh life, he just keeps his later working life active with confidence in his good work performance.

#### 5.1.4. Being Praised by Supervisors and Gaining Respect from Peers

Interviewees N and O, in particular, rationally approached the question of ageing. They said that ageing greatly affected health and the ability to carry on a normal life. Thus, they thought that it was the loss of health through ageing that may diminish the chances of older workers in the competitive labour market. However, interviewees N and O presented their strong points, as the other interviewees did.

Interviewee N said,

“My supervisor has praised my work. However, I now feel much more tired than when I was young. My work performance is similar to what it used to be”.

Interviewee O commented,

“After work, my muscles ache. When I was young, I slept well. I was all right. However, now I feel different. Although I sleep through the night, I ache the next morning. My back aches and I feel stiff every morning. However, I work well. I ignore the pain. Once I start working, I forget the pain. It’s nothing … They call me the Elder Sister here out of respect”.

Ageing can have a significant impact on their health and daily lives and cause them to lose employment opportunities in the labour market. However, nothing can prevent their active ageing. This could be generated from the recognition and respect of their peers who appreciate their good work performance.

Meanwhile, Interviewee N first boasted about being praised for work and then candidly confessed to feeling tired, whilst interviewee O firstly noted that his body ached after working, then emphasized his good work performance including Korean traditional food cooking skills by calling his pain “nothing”, since it can be forgotten when he starts working. Perhaps interviewee N identifies, from the point of view of her supervisor, a more significant capacity to work than her physical tiredness would suggest, while interviewee O tends to be more concerned with his pain than his pride as a result of working well, as the word “ache” seems to confirm. Two levels of hardship are shown in the words of these two older people. Older workers may have similar opinions about ageing and their work performance, but the intensity of their statements on physical decline and job performance may differ between individuals.

### 5.2. Considering Individual Work Ability of Active Older Workers Including Health

Interviewees require society to see individual health rather than the general health condition of older people in employment, because they assert that their good health is the critical factor influencing active ageing in comparison to other older people.

#### 5.2.1. “I Don’t Feel It Is Too Old to Teach”

Interviewee F, however, saw that weakness goes hand-in-hand with illness. He remarked,

“As people grow old, they age through and through, except for those who are extremely healthy. Once people age, they have conflicts, problems and illnesses”.

His negative perception of ageing influences his views on health and performance of older workers. In particular, he talks about “conflict” and “problems”. These words are negative but reflect that some people are indeed burdened with problems. He, however, suggests that it is only older people who are affected. However, regarding himself, he went on to say,

“I can teach students in schools but now there is no opportunity since I retired. I’m 76, but I don’t feel it is too old to teach—yet society does”.

He believed that his capacity to teach was no different, even now that he had retired. Thus, when he talks about other older people, he does not view them positively, but when he talks about his own capacity, he becomes positive, even though he too, is old. His work ability is summed up in the phrase, “I don’t feel it is too old to teach”. It is hard to reconcile his general perception of ageing and his self-assurance about his own work ability. One thing that can be assumed is that he formed his perceptions of ageing by generalizing older people as a group, whilst he specifically considers his own case, his professional teaching skills, as an individual exception to be viewed positively. In this sense, a specific approach to each older worker’s workability may be necessary to promote the active ageing in employment.

#### 5.2.2. “I’m Different from Other Older Workers”

The perception of interviewee J was very similar to that of interviewee F. She had the negative view that older workers are too narrow-minded to understand people unlike themselves. This makes it hard for older people to cooperate with younger workers. Believing that age made people slow in movement and language, she thought that full-time jobs were hard for older workers to do. However, interestingly, she also recorded an experience to the contrary:

“I’m different from other older workers. When I worked in the locker room of a golf club, I was very popular because of my brilliant work performance. All the customers and colleagues liked me very much”.

What she wanted to convey was that she herself had a pleasant work personality, despite her own view of other older workers. “I’m different” implies that most older workers have a negative perception, reflected from years of social prejudice as well as their own experience of bodily limitations, but she was confident of being exceptional; her own work performance was good. In particular, she compliments herself while pointing out that she is very popular. She believes that the reason for her popularity is her good workability, such as accountable customer service. The subjective belief for her good workability seems to make her work and earn money, as she wants to do and, as a whole, happy and active in her later life. This is the salient point to grasp.

#### 5.2.3. Individual Approach to Health beyond Chronological Ageing

Interviewee N, in her comments on ageing pointed to health:

“People cannot be healthy forever. Each person is also different in how far ageing affects their workability. However, I think that health is the aspect which affects active ageing the most”.

Her view that health is the most influential factor in active ageing and work ability may have been obtained through her experience with previous older colleagues. In particular, she points to

“The best worker in my opinion … [a] person who is 85 years old. She is a very funny lady”.

She does not introduce the older colleague by giving her age but describes her first as the best worker and then mentions how old she is. According to her experience, chronological ageing has no effect on the workability of employees. For this reason, she concentrated on health when she was talking about the perception of active ageing. However, she recognizes that people cannot be healthy forever. In her logic, when people grow older, their health is uncertain and the ability to work may be influenced by one’s state of health. This implies that she understands and accepts that ageing can negatively affect the work ability of older people, although she regards the extent of ageing, such as the state of their health, as different in different people.

### 5.3. Negative Social Prejudices for the Work Ability of Older People Have Been Obstacles toward Active Ageing

Social changes with renovated technologies were the causes of older people having difficulties in employment. Thus, in the perception of the interviewees, society judges older people based on their old age, which can be a description of older people, rather than acknowledging their good work performances, which can be a result of good health.

#### 5.3.1. “Society Goes Ahead So Fast That I Cannot Catch Up with It”

Uniquely, interviewee O described ageing as a matter of changing times, which impacted on his earlier experience of retirement.

“This generation does not forgive…Abacus calculation was my weapon. However, they use computers, machine … there is no space in the social system. Society goes ahead so fast that I cannot keep up with it”.

His perception of ageing was not related to physical health but to social changes that outpaced him as he grew older. He may have believed that active ageing had no effect on his physical condition but felt that the excessive speed of social developments caused him to regress in those areas where he used to work. In other words, his perception of ageing was not the decline of the physical body but the social pressure, which he could not cope with, to keep up with modern technologies such as computers. He does not blame society and the younger generation but considers that he is to blame for not updating his skills, in view of the rapid developments in the social system. In this sense, conversely, it can be assumed that he might want to find the hope of going back to work again when he updates his skills or develops some new ones. He blames himself, whom he can change, rather than criticizing the movement of society, which he can do nothing about.

#### 5.3.2. Society Does Not Acknowledge the Good Work Performance of Healthy Older People

Interviewee B, for her part, presented a radical perception of ageing:

“Ageing is just counting. I’m 75. Sometimes, if I work too hard, I feel tired as I used to do when I was young. Since I started working at 50 (for her children’s education), I don’t feel any difference in health, so I have no difficulty in working. There are lots of healthy older people. I’m one of them. They don’t feel old”.

As she emphasized “counting” when she described ageing, to her mind, chronological ageing would seem to be a matter of numbers rather than an important influence on health or workability. In addition, it may be said that she has an active outlook that can compare the health of older and younger people, although the health of older people tends to be poorer than the health of the young. The reason for the comparison can be found in her experience: in her workplace, the older people, as she claims to include herself, are healthy enough to be competitive. However, to some extent, she may not be sure that ageing is a matter of numbers; she does not say that they are not old but merely that they do not feel old, implying in this context that using the word “feeling” prevents it, somehow, from being a fact. The reason for this is found in the following extract:

“Society does not acknowledge it. The work performance of younger people is complimented thanks to their young age, but the good workability of older people cannot be recognized because of their old age, although older people work better sometimes. I have experienced very unfair situations”.

She seemed to feel bitter about society’s unfair treatment of older people because she had seen that older people can stay abreast of the social competition. As she saw it, it was clear that what had led to society’s negative prejudice was not connected with the work ability of older people, but society’s failure to acknowledge it. This means that older people can achieve active ageing not only by themselves, but also by the social structure support that acknowledges their workability in the labour market.

### 5.4. Society’s Negative Prejudice for Older Workers Could Be Overcome by Experience with Active Older Workers in Age-Friendly Working Environments

Once people meet and experience excellent older workers, employers and employees could have positive perceptions of older workers. However, the fair wages for older employees remained questionable in the labour market. Despite unfair treatment for older workers in the working places, interviewees appreciated the places where they worked genuinely with a happy mind, age-friendly working environments and methodical support for older employees.

#### 5.4.1. Reformed the Perception of Ageing in Challenging Working Places as They Experience Excellent Older Workers

Some interviewees harboured ageist social prejudices of their own. Interviewee G said that she did not feel that, in work performance, older workers were any different from younger ones, but before she took a job in later life and met older colleagues, she had herself been prejudiced against older workers.

“I’m 71. Actually, I thought older people did not work well. In contrast, they do work perfectly well. Older colleagues are very diligent and have good cooking skills. They are very good workers, even compared with younger employees. I think that the negative perception of ageing is formed by social prejudice”.

In particular, her words “even compared with younger employees” are significant in her perspective on ageing. Comparing older people’s and younger people’s ability to work may seem unfair and unreasonable, as interviewee G had negative prejudice over older colleagues before she worked with them. Such prejudice depends on an assumption that younger people are stronger than older people, but her experience that older workers “work perfectly well” demonstrates that the perspective on ageing can be influenced by individual experiences of working with older workers. Furthermore, the narrative of interviewee G suggests that society’s negative view of ageing can be changed to a positive one if more opportunities for younger and older people to work together can be developed.

Similar to interviewee G, interviewee H also implied that, in his experience, chronological ageing was not relevant in the labour market. This suggests that he may have shared this social prejudice against older people before he worked with them, as is confirmed by his stress on “but” in the sentence quoted below. If he had not been prejudiced, he would not have been astonished by the good workability of older people.

“I’m 72. There is an older worker who is 80, but works very well. Sometimes, I feel I’m a young man in comparison to this older one”.

The term “young man” seems to imply that he is the young man in not being as capable as his colleague. “Works very well” endorses this supposition. In other words, it can be assumed that his perception of ageing may have derived from his former milieu, where people had no positive views of ageing, but now he had reformed his perception of ageing in a new, challenging social setting as he reconstructed his later life.

#### 5.4.2. “I Would Like a Bigger Wage” over the Positive View on Older Workers

Interviewee I felt ashamed of growing older. The older he grew, the more he encountered discrimination in employment. It seems to him that ageing is a notion formed from nothing but social prejudice against older workers, which reinforces the negativism of employers towards them. However, he claimed,

“I don’t feel any difference in strength although I’ve some difficulty in learning English”.

Interviewee I felt ashamed to have been unemployed because of previous negative perceptions of older workers. In his current job, he had been hired thanks to his employer’s positive perception of his workability.

“I knew my boss before I came to work here. I used to come here every day with a lot of waste to sell for recycling which was collected from the street. At the time, my boss took a good view of my work. And then he suggested that I should work here. But my boss pays less and asks for more time at work than with younger workers. I am happy to work here. This job is so precious. But I would like a bigger wage”.

This case is somewhat different from those of other interviewees, who in the light of experience changed their views of older workers from negative to positive. Here, it is the employer who has changed his perception of older workers through getting to know interviewee I. However, this change does not extend to giving all older workers equal treatment and income in the labour market. From this older person’s perspective, the employer could not overcome social prejudice and had the same view of older workers as his younger employees, although he had developed a positive view of interviewee I. However, he had not offered him full-time work or pay equal with that of the younger workers. In this situation it is not possible to discern the reasons for the employer’s behaviour. The extract, “I am happy to work here. This job is so precious” reveals the conditions in the labour market with which interviewee I is faced; “the job is precious” means that the value of the job is something to treasure in an employment market where a negative social perspective does not favour older people. In this context, there is no doubt that older employees desire fair active ageing to have equal opportunities and payment alongside their good work performance. However, to achieve the hope of older workers, society and the government should build a social structure where older workers can prove their good workability against age discrimination in employment and will need to empower the workability of older people enough to obtain a job and reasonable income in the labour market if they dream of active ageing through employment.

#### 5.4.3. ‘I Don’t Feel Tired Here’ in Suitable Working Environments

Interviewee D first looked back at her previous work, where she achieved a good deal in the paint shop of a car factory. She is sure that she does not estimate her present achievement as any less. For example, she was promoted to supervisor in both jobs. At the age of 50, she did not feel tired, despite working hard until 9 pm each night to earn the maximum wage. In contrast, when she was past the age of 60 and tried working in an electronics factory, she felt too tired and consequently left the job. After this, she found an easier job in a food delivery bank. In her present workplace, she is working well and does not feel as tired

“…as in [her] previous work. In fact,”

she remarked,

“some jobs for older people can be physically hard work”.

A cooking job in a restaurant is not easy, but she said that this job was easier than her previous one. She points out that older workers often feel more tired than when they were younger. However, she does not feel that conditions in the restaurant are hard. This job seemed to suit her as she says, “I don’t feel tired here”. In her case, it seems that suitable working environments including her physical strength let her age actively in employment. In a few words, suitable working environments as well as health are mutually supported for active ageing in employment.

#### 5.4.4. Methodical Support of Society Can Generate Active Ageing Opportunities for Older People in a Workplace

Meanwhile, interviewee A faced generalizations on ageing with questions and doubts. From her perspective, physical ageing depends on the individuals and can be overcome by methodical support in the workplace. She accepted that most older people had health problems, such as poor eyesight, weakness and arthritis, simply because of their age, but she gave as an example the fact that she overcame her arthritis by using support equipment, such as special seating in her knitting workplace, which allowed her to work sitting down. However, she accounted for the popularity of older workers in the Senior Club business market by their professional standards, such as hers in knitting. In particular, however, she emphasized her ineffective competitiveness in the open labour market.

“At this age, where can I go? What can I do? Does society give jobs and money to older people?”

The advantage that the government provides through the Job Creation Act is, in her view, a space for her to work. In particular, her phrase, “Society gives jobs and money” conveys the social conditions that she finds in the labour market. The chance for older people to be taken on payrolls is the gift of society alone. In short, as she sees it, the one who decides on a job offer is not the older employee who wants to have a job, but wider society. In this sense, it shows the possibility that older employees are able to accomplish active ageing with the methodical support of society.

## 6. Discussion

Four themes were generated from the research. The first and second themes are regarding the driving force to make older people achieve active ageing. The third theme is related to the social barriers that preclude the economic participation of active older people. The fourth theme is the suggestions of interviewees regarding tackling the social barriers of active ageing. These four themes will be discussed in turn.

In Korean studies, the main reasons older people were working were an economic need and a joy or happiness in work [4,5,6]. However, it was difficult to find out what allows older people to realize their desire to work. The first and second theme present the driving force for it.

In the first theme, research participants have confidence in their good work performance, with professional efforts in their highly skilled area, while overcoming their weakened physical strength and deteriorating health. Furthermore, they had a challenging attitude to their ageing, and they attempted to demonstrate good work performance to the extent to which their colleagues praise them, and their supervisors show their respect to them. In this theory, the motive power is likely to be their confidence in their good work performance and challenging working attitude to ageing. This aspect supports the approach of the OECD, which defines active ageing as the “capacity of people, as they grow older, to lead productive lives in the society and economy” [13]. In addition, being praised by supervisors and gaining respect from peers, which might be resulted from the good work performance of older workers, can be a significant driving force such that the research participants maintain their active economic role in society.

On the other hand, in the research of the Korea Institute for Health and Social Affairs [6], maintaining health was the reason to work. However, in the second theme, health is the work ability which is a condition of older people being offered a job. The interviewees believed that their capacity is influenced not by chronological ageing, but individual health level. Meanwhile, they regard that they are different from other older people, who are getting weaker and more ill, because they are healthy enough to work well and have sufficient work ability to perform their jobs. In short, they suppose that active ageing in their working life depends on individual working ability levels, including health.

In this respect, the first and second themes accompany the aspect that older people’s efforts to remain in their social role can contribute to the active ageing, although the intensity of participation in active ageing depends on an individual’s “life course and life situation” [18,19,20].

This theme is supported by Wongsala, Anbäcken and Rosendahl’s [25] active ageing research, which applies factors of the WHO to discuss that the social participation of seniors provides older people with respect, meaningful life and social support.

In the third theme, the older interviewees desired to work and maintain their economic position in the labour market. However, they were desperate that society does not accept their needs to work because of society’s negative prejudice toward older people and does not have enough patience to wait for them to catch up to modern society with updated technology. In addition, they felt disgraced that society is not aware of the decent work ability of older people. They believed that society does not evaluate older people based on their workability but rather it only judges older people on the chronological age in the labour market. In other words, in their perspective, society is not likely to cooperate with older people who desire to participate in active ageing.

The significant third theme supports Walker’s [9] assertion that active ageing is a social responsibility because the barriers to active ageing cannot be tackled only by individual efforts. This research interviewees seemed to understand the social structure stated in the perception of the Walker [9]. However, social participation should be optional for older people. As Taylor [21] pointed out, active ageing should not overlook the benefit of retirement to provide older people with opportunities to live a life they hope for in their later life, while getting far away from economic activity for the cost of living in a secure pension system. Alongside Taylor’s [21] concern that stressing social participation might be a reason for saving social welfare costs for older people, Zaidi and Um [24] show the high economic activity level of active ageing index in Asian countries, in comparison to European countries, might be influenced by the low pension income in Asian countries. Additionally, in Korean active ageing index research, it was supposed that the higher employment level of Korea might be caused by low pension incomes. In this context, it is suggested that the social participation of active ageing should be encouraged and promoted in the assurance of social security for older people.

In the fourth theme, even research participants could not perceive the good workability of older people before they met excellent older workers, even though they are older workers. Employers were the same as the older interviewees. Once an employer experienced an older workers’ good work performance, they positively changed their attitude and mindset towards older employees. However, according to their work experiences, it was very difficult for the employers to show equal treatment to older workers in wages. Thus, interviewees wished for a fair wage system, equal to the younger workers. Additionally, they hoped that the government and employers would provide more job opportunities and methodical support for their work. In their belief, active ageing in employment can be accomplished by not only the efforts of older people, but also social support to provide older people good working environments—as Walker [9] pointed out, the employment of older people is a social concern. Moreover, Taylor [21] was concerned that society abandons the risk of working places, which older employees might encounter. In this sense, a suitable working environment for older people should be considered by the government and employers.

Havighurst and Albrecht [10] (1953) found that the more that older people participate in economic activity, the happier they are with life. In this perspective Korea has developed the Job Creation Act for active ageing in the approach of productive welfare. However, Gi [29] pointed out that the Korean government has mostly stressed economic participation to save the social welfare cost of older people. She asserts that voluntary activity should also be encouraged as social participation for active ageing.

Moreover, in Korean studies, health is much more significant in active ageing than social participation and living security among the active ageing factors composed by the WHO. Hyun [27] and Oh [28] agree that health is significant for life satisfaction in their research. In this context, it can be suggested that the government should develop policies to maintain and improve the health of older people as well as developing policies and practices for the employment of older people.

In the meantime, Heo’s [30] and Byun [31]’s research indicates that the more an aged person’s income status is secured, the more they participate in social and economic activity for active ageing. It implies that the government should ensure income security for older people along with improving the health of older people for active ageing.

Additionally, active ageing in daily life should be respected, as Clarke and Warren [22] found that older people regard ordinary needs, including health, deeds for daily life, events of the years and interaction with family and friends, as active ageing.

Nevertheless, this has clear implications for policy with respect to active ageing in economic activity. This biographical research finding ensures that older people strongly desire to work for their life and happiness, as it was also shown in the Korea Institute for Health and Social Affairs [6]. They are very proud of and content with their working life, despite the negative social prejudice towards older workers in the labour market.

## 7. Conclusions

This article aimed to understand and interpret the perspectives and experiences of employed older people on active ageing in their biographical contexts. From the research results, it was found that older people had confidence in their good workability and challenging attitude at work. In short, their confidence to work and their belief on the individual good workability of older workers in the labour market led older people to achieve active ageing in employment. However, they believed that social barriers, including a negative social prejudice regarding the working ability of older people, have obstructed their working life. Thus, they wished that the opportunities to show their good workability should be extended and relevant working environments would be developed for older people to accomplish active ageing in their later working life.

To sum up, it can be recommended that society should focus on active ageing, which can promote older people to undertake social participation including economic activity as well as good health and living security in balance. As the WHO [15] pointed out, active ageing helps older people to “realize their potential for physical, social, and mental well-being throughout their life course and to participate in society according to their needs, desires and capacities, while providing them with adequate protection, security and care when they require assistance”.

However, the policy implication of this interpretive research has a limitation on policy practices. Individual voices cannot be a representative and generalized view. In this sense, for the policy suggestion for active ageing, qualitative research with a bigger sample to ensure the theory’s generalization can be recommended. However, this biographical narrative research still has significance for the policy suggestion in active ageing. As this research presented above, Fraser [33] remarks:

“The identities and needs that the social welfare system creates for its recipients have always interpreted their identities and needs”.

In this context, the voices of these research participants, obtained using the biographical narrative approach, are significant to understand the needs of older people for policy suggestions regarding active ageing in employment.

Despite this qualitative research’s relevance for active ageing studies, for further research, it is recommended that a diverse, balanced and relevant theoretical sampling, including gender and education level, be considered. It can be expected that more precise theoretical sampling will provide more relevant and significant research findings and interpretations from research participants in active ageing studies. Additionally, for the active ageing conceptualization, the inclusion of a younger group of people in sampling to gain their perspectives and experiences regarding working with older people is recommended. The view and experiences of a younger group of people can provide a broadened and extended concept of active ageing in employment to the academic and policy approach.

In conclusion, the interpreted words of older people in terms of active ageing in their later working life presents their need to have social recognition for their good workability and tackle social barriers by the government and society providing employers and other employees with experiences to meet excellent older workers and social supports, including age-friendly working environments for their active ageing.

## Figures and Tables

**Table 1:** A summary of the personal data in the research group.

Pseudonym of Interviewee	Age	Gender	Place	Type of Business	Family	Education	Income Resource per Month
Interviewee A	75	F	Junju Medium-sized city	Knitting business and health food business	Husband, 2 sons and 2 daughters	Primary school	USD 347 from children, USD 277 from job
Interviewee B	75	F	Wanju Rural area	Food delivery centre	Husband, 4 sons and 1 daughter	No school	USD 277 from job
Interviewee C	66	F	Kwangju Big city	Food delivery centre	Husband, 3 sons	Primary 1	USD 277 from job
Interviewee D	66	F	Kwangju Big city	Food delivery centre	Husband, 1 son and 2 daughters	No education	USD 277 from job, USD 62 from the basic old age pension
Interviewee E	69	F	Wanju Rural area	Food delivery centre	Husband, 2 sons and 1 daughter	No education	USD 277from job, USD 104 from the basic old age pension
Interviewee F	76	M	Inchon Big city	Occasional primary school teacher and parcel delivery	Wife, 2 sons and 1 daughter	PhD	USD 138 from job, USD 277 from national pension
Interviewee G	71	F	Inchon Big city	Restaurant	4 sons	High (secondary) school	USD 347from job
Interviewee H	70	M	Seoul	Parcel delivery	1 son and 1 daughter	Primary school	USD 624 from job
Interviewee I	79	M	Namwon	Recycling centre	2 sons and 1 daughter	Primary school	USD 208 from job, USD 104 from the basic old age pension
Interviewee J	66	F	Yeoju	Knitting work	New husband, 2 sons	High school	USD 138 from job, property assets
Interviewee K	68	F	Seongnam city	Cleaner	2 sons and 2 daughters	No education	USD 555 from job
Interviewee L	79	F	Yeoju	Knitting work	1 son and 3 daughters	Primary school	USD 138 from job
Interviewee M	72	M	Seoul	Parcel delivery	Wife, 1 son and 1 daughter	High school	USD 138from national pension, USD 138from job
Interviewee N	77	F	Gapyung	Food bank (health foods)	2 sons and 2 daughters	University	Much income from assets, USD 138 from job
Interviewee O	68	M	Seongnam	Security guard	3 daughters	University	Wife’s income, USD 555 from job

**Table 2:** Research findings in structure.

Research Questions	Theme	Sub-Theme
1. What brings you economic activity in active ageing?	5.1. The confidence of the employed older people on work ability and their challenging attitude at work led them to active ageing in employment.	5.1.1. Deteriorating health but professional work
		5.1.2. Highly skilled work in her job area
		5.1.3. His sense of challenge to work
		5.1.4. “Praised” by supervisors and “respect” from peers
	5.2. Considering individual work ability of active older workers including health	5.2.1. “I don’t feel it is too old to teach”.
		5.2.2. “I’m different from other older workers.
		5.2.3. Individual approach to health beyond chronological ageing
2. What tackles your economic activity in active ageing?	5.3. Negative social prejudices for the work ability of older people have been obstacles towards active ageing	5.3.1. “Society goes ahead so fast that I cannot catch up with it”
		5.3.2. Society does not acknowledge the good work performance of healthy older people
3. What do you suggest for tackling the barriers of economic activity in active ageing?	5.4. Society’s negative prejudice for older workers could be overcome by experience with active older workers in age-friendly working environments.	5.4.1. Reformed the perception of ageing in challenging working places as they experience excellent older workers
		5.4.2. “I would like a bigger wage” over the positive view on older workers
		5.4.3. “I don’t feel tired here” in suitable working environments.
		5.4.4. Methodical support of society can generate active ageing opportunities for older people in a workplace.

## Data Availability

Not applicable.
